# The incidence of bronchiectasis in chronic obstructive pulmonary disease

**DOI:** 10.1515/med-2022-0599

**Published:** 2022-12-06

**Authors:** Hsueh-Yi Lu, Kuang-Ming Liao

**Affiliations:** Department of Industrial Engineering and Management, National Yunlin University of Science and Technology, Yun-Lin, Taiwan; Department of Internal Medicine, Chi Mei Medical Center, Chiali, Taiwan

**Keywords:** chronic obstructive pulmonary disease, bronchiectasis, incidence.

## Abstract

Bronchiectasis is a common comorbidity in chronic obstructive pulmonary disease (COPD). There are limited data regarding the incidence of bronchiectasis in COPD. The purpose of the study was to use a nationwide database to evaluate the incidence of bronchiectasis in COPD in Taiwan. We used a cohort of 2,000,000 individuals followed from 2005 to 2018. Patients with COPD diagnosed between January 1, 2011, and December 31, 2017, were selected, and those with bronchiectasis before COPD were excluded. In total, 134,366 patients with COPD were enrolled, and propensity score matching was used to ensure homogeneity of baseline characteristics between the COPD and non-COPD groups. The incidence rate of bronchiectasis was higher in the COPD group than in the non-COPD group (87.83 vs 69.80 per 10,000 person-years). The adjusted hazard ratio (1.9; 95% confidence interval 1.75–2.05; *P* < 0.001) of bronchiectasis indicated that the risk of bronchiectasis was 1.9 times higher for patients with COPD than for patients without COPD. In the COPD group, the age-stratified incidence rates of bronchiectasis increased with age (55.01, 80.92, 101.52, and 105.23 for 40–49, 50–59, 60–69, and over 70 years, respectively). The incidence of bronchiectasis was higher in patients with COPD than in the general population, the risk of bronchiectasis increased with age in COPD, and post-tuberculosis status was an important risk factor for bronchiectasis.

## 1 Introduction

Chronic obstructive pulmonary disease (COPD) is a chronic and systemic inflammatory disease [1]. It is characterized by airflow obstruction that is not fully reversible, and slow disease progression leads to major morbidity and mortality worldwide [2]. Patients with COPD had multiple comorbidities. Bronchiectasis is one of the common comorbidities in COPD [3]. Bronchiectasis was also common in patients with COPD and may be a specific phenotype in COPD. Bronchiectasis was associated with emphysema and increased airflow obstruction, severity of COPD, and mortality [4]. Bronchiectasis was commonly detected and associated with disease severity in patients with COPD–obstructive sleep apnea overlap syndrome. Bronchiectasis was related to more severe hypoxemia and increased systemic inflammation [5]. A systematic review and meta-analysis showed that bronchiectasis was associated with acute exacerbation, airflow obstruction, presence of potential pathogenic bacteria, and mortality in patients with COPD [6]. Authors found some factors associated with bronchiectasis in patients with COPD, which included severe airflow obstruction, presence of potential pathogenic bacteria from sputum, and hospitalizations for exacerbations in the previous year [7].

Previous studies demonstrated the impact of bronchiectasis on COPD in multiple directions and suggested that bronchiectasis was one of the pathological phenotypes in COPD and may predict prognosis [8]. Bronchiectasis is irreversible airway dilatation diagnosed on imaging [9] and a clinical presentation of cough, phlegm, and recurrent airway infection. The clinical presentation of bronchiectasis inevitably overlaps with that of COPD, and clinical states of bronchiectasis–COPD overlap syndrome (BCOS) are observed [10]. One study enrolled 133 patients with COPD at one hospital to determine the incidence, clinical characteristics, and related factors of bronchiectasis in COPD [11].

There are no nationwide data regarding the incidence of bronchiectasis in COPD. The aim of our study was to use a nationwide database to evaluate the incidence of bronchiectasis in COPD in Taiwan.

## 2 Methods

### 2.1 Data sources

The Ministry of Health and Welfare in Taiwan has established a nationwide-coverage health care plan called the National Health Insurance (NHI) program, which includes 97% of healthcare providers and covers approximately 99% of the 23 million people living in Taiwan. The NHI Research Database (NHIRD) is a collection of health information for academic research that was initiated by the Data Science Center of the Ministry of Health and Welfare to improve the quality of public health decision-making and to enhance well-being. The NHIRD is one of the largest scale administrative health care databases worldwide. It contains all the inpatient and outpatient registration and claim data of the NHI program. The database includes patients’ demographic characteristics, disease-diagnostic and surgery-operation codes (based on the International Classification of Diseases, Ninth Revision, Clinical Modification [ICD-9-CM]), prescription data, and medical expenditures. In this study, we used a longitudinal dataset from the NHIRD containing a cohort of 2,000,000 randomly selected enrollees followed retrospectively from 2005 to 2018. All research data were processed and computed at the Data Science Center with strictly regulated data deidentification. The personal information in the dataset was deidentified, and no statistically significant differences in age, sex, and health care cost distributions were present among the selected subjects.

### 2.2 Patients

**Figure 1:**
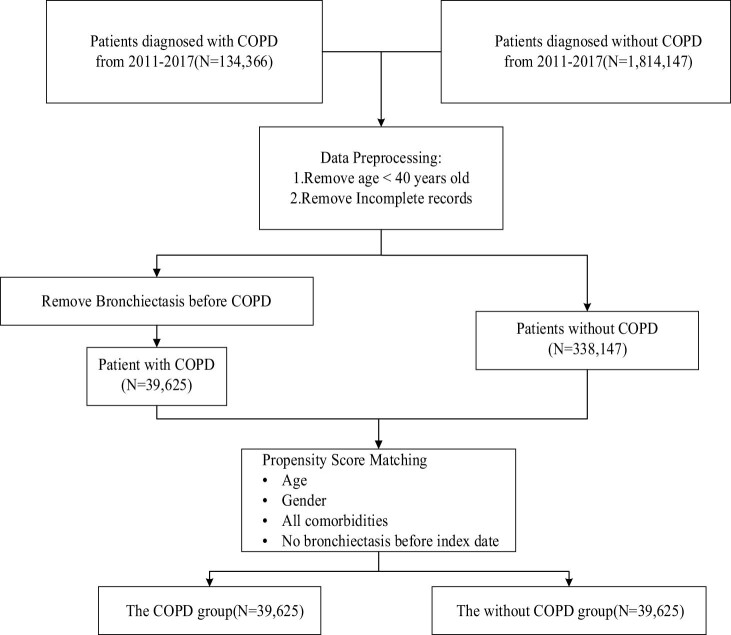
Flowchart of subject enrollment.

The COPD subjects were patients diagnosed with ICD-9-CM codes 490-492 and 496 between January 1, 2011, and December 31, 2017. To ensure that the patients were COPD patients, the subjects were required to have at least three outpatient visits or one inpatient admission with a main diagnosis of COPD in the NHIRD records. The earliest date of the third visit or inpatient admission was designated as the index date to investigate the risk of bronchiectasis. In total, 134,366 patients with COPD qualified preliminarily before exclusion filtering (Figure 1). The exclusion criteria were age less than 40 years, asthma, bronchiectasis before the index date, or incomplete records.

### 2.3 Propensity score matching

The control group consisted of subjects randomly selected from among hospitalized patients without COPD diagnosis in the database. To eliminate baseline differences between COPD and non-COPD patients, matched controls were used by applying propensity score matching. The propensity score was estimated from a probability function based on a multivariable logistic regression model to ensure the homogeneity of baseline characteristics and reduce selection bias related to covariates between the COPD and non-COPD groups [12]. Covariates included age, sex, diabetes mellitus, hyperlipidemia, hypertension, cerebrovascular diseases, end-stage renal disease, chronic liver disease, hepatitis B, hepatitis C, and tuberculosis. The control subjects were matched and selected with a propensity score of ±0.05 standard deviation (SD) at a 1:1 ratio. During one-on-one matching process, non-COPD patients having bronchiectasis before the index date of matched one were excluded.

### 2.4 Outcomes and comorbidities

To estimate the risk of bronchiectasis, the patients were followed until bronchiectasis occurred (ICD-9-CM code 494), death, withdrawal from the NHI, or the end of 2017. Comorbidities including diabetes mellitus (ICD-9-CM code 250), hyperlipidemia (ICD-9-CM code 272), hypertension (ICD-9-CM code 401–405), cerebrovascular disease (ICD-9-CM code 430–438), end-stage renal disease (ICD-9-CM code 585), chronic liver disease (including cirrhosis, ICD-9-CM code 571.5, and 571.6), hepatitis B (ICD-9-CM code V02.61, 070.20, 070.22, 070.30, and 070.32), hepatitis C (ICD-9-CM code V02.62, 070.41, 070.44, 070.51, and 070.54), other chronic hepatitis (ICD-9-CM code 571.40, 571.41, 571.49, 571.8, and 571.9), and tuberculosis (ICD-9-CM codes 010, 011, 012, 013, 014, 015, 016, 017, and 018) were identified from the outpatient and inpatient records 1 year before the index day.

### 2.5 Statistical analysis

Demographic and comorbidity variables for patients with and without COPD are expressed as frequencies (percentages) or mean values (±SDs) and were compared using chi-square tests and Student’s *t* tests. The demographic characteristics included sex and age (stratification into 40–49, 50–59, 60–69, and over 70 years). Cumulative incidence curves for bronchiectasis were plotted using the Kaplan–Meier method, and the differences in curves between the with and without COPD groups were tested by a log-rank test. The incidence rate of bronchiectasis was estimated using the total number of bronchiectasis events divided by the total follow-up period (per 10,000 person-years). A Cox proportional hazards model was used to measure the main effect of comorbidities for patients with COPD at the time of bronchiectasis occurrence. Hazard ratios (HRs) and their 95% confidence intervals (CIs) were estimated by Cox regression. Variables with significant values in the univariable model were further examined in the Cox regression model. All statistical tests were two-sided, and a *P*-value of 0.05 was considered significant. The statistical analyses were performed with SPSS (version 15; SPSS, Inc., Chicago, IL, USA).


**Ethics statement:** This study was reviewed by the Institutional Review Board (IRB) of the Chi Mei Medical Center, Taiwan (IRB no. 10906-E01). Informed consent was waived by the approving IRB. All personal-related information was deidentified in the dataset, with anonymity strictly maintained by the Data Science Center, Ministry of Health and Welfare.

## 3 Results

### 3.1 Patient characteristics

A total of 39,625 eligible patients with COPD were identified in the study group (Figure 1). Propensity scores were calculated for covariates associated with bronchiectasis for all patients. After propensity score matching, the control group (non-COPD) comprised 39,625 comparable patients. The study group had 1,704 (4.3%) patients with bronchiectasis, and the control group had 933 (2.4%) patients (Table 1). The comparisons of basic characteristics between the two groups (COPD vs non-COPD) are shown in Table 1. The variables such as age, sex, and comorbidities were evenly distributed between the two groups, thereby increasing between-group comparability.

**Table 1:** Demographic characteristics and comorbidities of patients with and without COPD

Variables	COPD (*n* = 39,625)	Without-COPD (*n* = 39,625)	Standardized mean difference
Age (%)			
40–49	4,518 (11.4)	3,737 (9.4)	
50–59	6,618 (16.7)	6,831 (17.2)	
60–69	8,129 (20.5)	8,681 (21.9)	
≥70	20,360 (51.4)	20,376 (51.4)	
Mean (±SD)	68.44 (12.76)	68.44 (12.75)	0.001
Gender (%)			
Male	24,954 (63)	24,970 (63)	0.001
Female	14,671 (37)	14,655 (37)	
Comorbidities (%)			
Diabetes mellitus	13,827 (34.9)	13,791 (34.8)	0.002
Hypertension	27,008 (68.2)	27,077 (68.3)	0.004
Other chronic hepatitis	1,287 (3.2)	1,245 (3.1)	0.006
Hyperlipidemia	15,536 (39.2)	15,552 (39.2)	0.001
Cerebrovascular disease	3,542 (8.9)	3,521 (8.9)	0.002
End-stage renal disease	5,063 (12.8)	5,014 (12.7)	0.004
Hepatitis B	2,136 (5.4)	2,124 (5.4)	0.001
Hepatitis C	1,592 (4)	1,572 (4)	0.003
Tuberculosis	59 (0.1)	38 (0.1)	0.015
**Outcomes (%)**			
Bronchiectasis	1,704 (4.3)	933 (2.4)	

### 3.2 Incidence of bronchiectasis

In Table 2, the overall incidence rate of bronchiectasis was higher in the COPD group than in the non-COPD group (87.83 vs 69.80 per 10,000 person-years). The adjusted hazard ratio (aHR 1.9; 95% CI 1.75–2.05; *P* < 0.001) of bronchiectasis indicated that the risk of bronchiectasis was 1.9 times higher for patients with COPD than for the non-COPD group. This relationship was further characterized by examining the association between age, sex, and comorbidities. In the COPD group, the age-stratified incidence rates of bronchiectasis increased with age (55.01, 80.92, 101.52, and 105.23 for 40–49, 50–59, 60–69, and over 70 years, respectively). These incidence rates of bronchiectasis also increased with age in the non-COPD group. In the Cox regression analysis, age, sex, and comorbidities were associated with higher risks of bronchiectasis in the COPD group than in the non-COPD group (Table 2). For example, for patients with hepatitis B, the aHR of bronchiectasis in the COPD group was 2.30 (95% CI 1.62–3.25; *P* < 0.001) times that of the non-COPD group.

**Table 2:** Incidence of bronchiectasis in patients with and without COPD

Characteristics	COPD (*N* = 39,625)			Non-COPD (*N* = 39,625)			aHR (95% CI)	*P*-value
	Event	TFP(PY)	IR	Event	TFP(PY)	IR		
Bronchiectasis	1,704	194,014	87.83	933	133,660	69.80	1.90 (1.75–2.05)	<0.001
Age								
40–49	113	20,542	55.01	20	20,128	9.94	4.75 (2.95–7.64)	<0.001
50–59	240	29,658	80.92	58	39,031	14.86	4.46 (3.35–5.94)	<0.001
60–69	369	36,348	101.52	151	49,733	30.36	2.72 (2.25–3.29)	<0.001
70 and above	982	93,324	105.23	704	117,595	59.87	1.45 (1.32–1.6)	<0.001
Gender								
Male	1,074	113,894	94.30	611	142,384	42.91	1.83 (1.65–2.02)	<0.001
Female	630	65,978	95.49	322	84,104	38.29	2.02 (1.77–2.31)	<0.001
Diabetes mellitus								
Yes	589	62,961	93.55	321	80,788	39.73	1.91 (1.67–2.19)	<0.001
No	1,115	116,911	95.37	612	145,699	42.00	1.89 (1.71–2.08)	
Hypertension								
Yes	1,140	123,548	92.27	709	157,746	44.95	1.68 (1.53–1.84)	<0.001
No	564	56,324	100.14	224	68,742	32.59	2.58 (2.21–3.01)	<0.001
Other chronic hepatitis								
Yes	63	5848.04	107.73	33	7,272	45.38	1.93 (1.27–2.95)	0.002
No	1,641	174,024	94.30	900	219,216	41.06	1.89 (1.75–2.05)	<0.001
Hyperlipidemia								
Yes	628	69,722	90.07	317	91,020	34.83	2.08 (1.12–2.38)	<0.001
No	1,076	110,150	97.68	616	135,468	45.47	1.8 (1.63–1.99)	<0.001
Cerebrovascular disease								
Yes	156	15,930	97.93	109	20,393	53.45	1.47 (1.15–1.88)	0.002
No	1,548	163,943	94.42	824	206,095	39.98	1.95 (1.79–2.12)	<0.001
End-stage renal disease								
Yes	209	23,203	90.07	130	29,431	44.17	1.67 (1.34–2.08)	<0.001
No	1,495	156,669	95.42	803	197,057	40.75	1.93 (1.77–2.1)	<0.001
Hepatitis B								
Yes	101	9,565	105.6	46	12,343	37.27	2.30 (1.62–3.25)	< 0.001
No	1,603	170,308	94.12	887	214,145	41.42	1.88 (1.73–2.04)	< 0.001
Hepatitis C								
Yes	76	7,174	105.94	43	9,172	46.88	1.83 (1.26–2.66)	0.001
No	1,628	172,699	94.27	890	217,316	40.95	1.9 (1.75–2.06)	<0.001
Tuberculosis								
Yes	7	243	288.05	10	173	578.03	0.42 (0.16–1.1)	0.077
No	1,697	179,629	94.47	923	226,315	40.78	1.91 (1.76–2.07)	<0.001

Abbreviations: COPD, chronic obstructive pulmonary disease; TFP, total follow-up period; PY, per 10,000 person-years; IR, incident rate per 10,000 person-years; aHR, adjusted hazard ratio; CI, confidence interval. Risk factors with *P*-value < 0.05 (age and sex) in the univariate analysis were used as adjusted covariates in the multivariate Cox regression.

### 3.3 Comorbidities and bronchiectasis

The association between comorbidities and bronchiectasis occurring in patients with COPD was further investigated (Table 3). The baseline group included patients with COPD with no comorbidities for comparison to those with various comorbidities. Significantly increased risks of bronchiectasis were found for patients with only tuberculosis (aHR 6.32; *P* < 0.001). Patients with COPD with exactly one, two, or three comorbidities were found to have significant risks of bronchiectasis (aHR 1.23, 1.20 and 1.18; *P* < 0.001) when compared to those with no comorbidity.

**Table 3:** Impact of comorbidities on bronchiectasis among patients with COPD

Comorbidity	Number	Event	aHR (95% CI)	*P*-value
No comorbidity	5,996 (15.1)	235 (3.9)	—	—
Diabetes mellitus only	700 (1.8)	39 (5.6)	1.26 (0.92–1.71)	0.15
Hypertension only	6,520 (16.5)	282 (4.3)	1.30 (1.13–1.495)	<0.001
Other chronic hepatitis only	49 (0.1)	3 (6.1)	0.96 (0.31–2.99)	0.94
Hyperlipidemia only	1,368 (3.5)	66 (4.8)	1.15 (0.91–1.45)	0.25
Cerebrovascular disease only	199 (0.5)	10 (5)	1.53 (0.94–2.49)	0.09
End-stage renal disease only	206 (0.5)	7 (3.4)	0.77 (0.4–1.49)	0.43
Hepatitis B only	199 (0.5)	11 (5.5)	1.3 (0.76–2.21)	0.34
Hepatitis C only	75 (0.2)	3 (0.04)	1.21 (0.5–2.9)	0.67
Tuberculosis only	12 (0.03)	3 (0.25)	6.32 (2.03–19.69)	0.001
≥1 comorbidity	32,989 (83.3)	1,436 (4.4)	1.23 (1.1–1.38)	<0.001
≥2 comorbidities	22,660 (57.2)	950 (4.2)	1.20 (1.06–1.35)	0.002
≥3 comorbidities	11,950 (30.2)	497 (4.2)	1.18 (1.04–1.34)	0.01

Abbreviations: IR, incident rate (event/number); aHR, adjusted hazard ratio. Risk factors with *P*-value < 0.05 (age and sex) in the univariate analysis were used as adjusted covariates in the multivariate Cox regression.

### 3.4 Cumulative incidence rates of bronchiectasis

**Figure 2:**
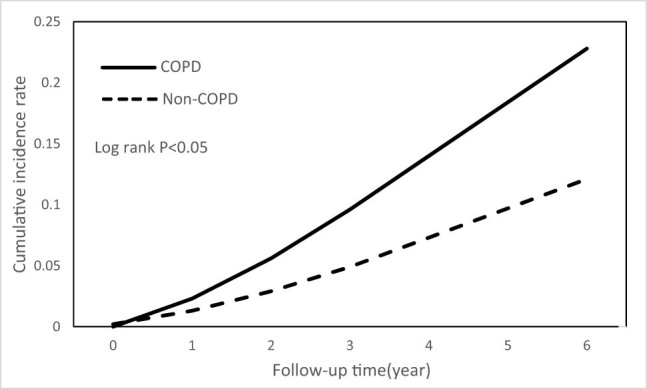
Cumulative incidence of bronchiectasis in patients with and without COPD.

**Figure 3:**
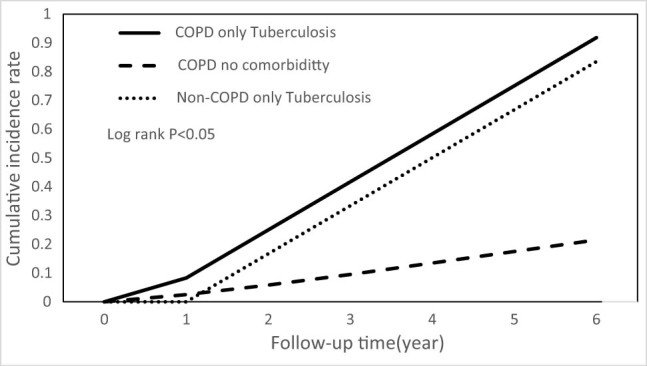
Cumulative incidence of bronchiectasis in patients with only tuberculosis.

Cumulative incidence curves estimating the occurrences of bronchiectasis over time showed significant differences (*P* < 0.05, by log-rank test) between the COPD and non-COPD groups (Figure 2). In Figure 3, patients with COPD with only comorbid tuberculosis had a significantly higher risk of bronchiectasis than patients with COPD with no comorbidity and non-COPD patients with only comorbid tuberculosis (*P* < 0.05, by log-rank test).

## 4 Discussion

The strengths of the study were to use a nationwide database to evaluate the incidence of bronchiectasis in COPD and related factors of bronchiectasis in patients with COPD, to identify the clinical characteristics of bronchiectasis. In our study, the incidence rate of bronchiectasis in COPD per 10,000 person-years was 87.83, and the aHR was 1.9 compared with patients without COPD. The incidence rate of bronchiectasis in COPD increased with age and was 105.23 per 10,000 person-years in individuals aged more than 70 years. The incidence rate of bronchiectasis was similar in male and female patients with COPD.

### 4.1 Aging and bronchiectasis in COPD

Previous studies have shown that the prevalence of bronchiectasis increases with age in the general population [13,14]. Quint et al. [13] used the Clinical Practice Research Datalink database to survey the incidence and prevalence of bronchiectasis in the UK between 2004 and 2013 and found prevalence of 35.17 females per 100,000 person-years in 2013 and 26.92 males per 100, 000 person-years in 2013. In our non-COPD population, the incidence of bronchiectasis was higher than that in Quint’s study because we followed patients until 2018 and the population comprised more older patients, and the incidence of bronchiectasis was associated with aging.

### 4.2 Sex and bronchiectasis in COPD

Previous validation cohorts found differences in bronchiectasis between women and men, being more common in women than in men [15]. Patients with COPD are found to have bronchiectasis on computed tomography, and similar frequencies are reported in patients with severe or uncontrolled asthma [16]. In our COPD cohort, the incidence of bronchiectasis was similar in women and men. Bronchiectasis results from a wide range of causes and is associated with other comorbidities, including asthma and COPD [7,17,18]. Asthma is common in bronchiectasis, and women are more likely to have asthma. Our COPD cohort did not include patients with asthma, and male patients were more common, making the incidence of bronchiectasis similar in women and men.

### 4.3 Comorbidities in patients with COPD and bronchiectasis

Bellelli et al. [19] analyzed six European databases of adult outpatients with bronchiectasis, and common comorbidities included COPD, rheumatologic disease, chronic renal failure, and diabetes mellitus. Chalmers et al. [15] found that chronic cardiac disease, cerebrovascular disease, and chronic renal failure were common comorbidities. A study of four European centers showed that the most common comorbidity in patients with bronchiectasis was gastroesophageal reflux [19]. The comorbidities in patients with COPD and bronchiectasis were heterogeneous and depended on the enrolled populations, study periods, definition, and database.

Our study excluded asthma patients and found that the most common comorbidities were hypertension, dyslipidemia, and diabetes in patients with COPD and bronchiectasis. These comorbidities were common in the COPD population [20] and observed in the subgroup with bronchiectasis. Previous studies found that patients with bronchiectasis had a range of comorbidities similar to those with COPD [21].

Biological mechanisms leading to bronchiectasis may have a role in the development of comorbidities [22]. COPD is a systemic inflammatory disease. Bronchiectasis also increased lung inflammation due to the pathology of repeated infection in the small airways. COPD and bronchiectasis have similar comorbidities and chronic inflammatory lung status. Patients with bronchiectasis had similar increases in comorbidities in COPD, such as increased arterial stiffness, reduced 6-min walk distance, low physical activity, and osteoporosis [22,23].

### 4.4 Tuberculosis and bronchiectasis in COPD

The etiology of bronchiectasis showed significant discrepancies in previous reports. An analysis of seven databases of bronchiectasis studies [24] found that the most common etiology was being in the post-infection period (20%), followed by COPD (15%), and idiopathic bronchiectasis accounted for 40% of patients. However, data from 106 patients in the USA [25] reported that the etiology of bronchiectasis was most often due to immune dysregulation, including autoimmune disease (*n* = 33, 31.1%), immunodeficiency (*n* = 18, 17%), hematologic malignancy (*n* = 15, 14.2%), and α1-antitrypsin deficiency (11.3%), only 7% of bronchiectasis patients were idiopathic. In another cohort of 15,729 adult patients from the Chang Gung Research Database in Taiwan [26], the most common etiology of bronchiectasis was idiopathy (32%), followed by post-pneumonia status (24%), COPD (14%), and post-tuberculosis status (12%).

Race and ethnicity play a major role in the etiology of bronchiectasis. Cystic fibrosis is a common autosomal recessive inherited disorder and etiology of bronchiectasis among Caucasians, but it is rare in Asia, with an incidence of approximately 1 in 350,000 in the Japanese population [27]. Only ten Taiwanese patients with cystic fibrosis have been reported [28,29]. Tuberculosis is common in Asia, and the incidence of TB decreased in Taiwan from 72 cases per 100,000 person-years in 2005 to 41 cases per 100,000 person-years in 2017 [30]. In the past, the prevalence of tuberculosis was high in Taiwan, and patients who were post-tuberculosis infection had an increased risk of bronchiectasis. In our study, patients with post-tuberculosis infection status had an increased risk of bronchiectasis, with an aHR of 6.32. In Asia, the prevalence of bronchiectasis is higher than that in Western countries [31], and post-tuberculosis status is one of the important predictors of bronchiectasis [32]. Recently, Choi et al. [33] used a database to conduct a national cohort study in Korea and found that tuberculosis control was associated with a decreasing incidence of bronchiectasis in South Korea. However, the authors did not provide an HR for tuberculosis in bronchiectasis, and future work may survey the etiology and influence of bronchiectasis.

### 4.5 Limitations

There are some limitations in our study. Our database did not include computed tomography data, and the diagnosis of bronchiectasis was based on clinical physician diagnosis. The type and severity of bronchiectasis could not be identified. In addition, pulmonary function tests were lacking in our database. Except for age, sex, and comorbidities, other possible confounding factors were not considered in this research. These limitations were inherited due to database restrictions for the NHIRD.

## 5 Conclusion

We found that the incidence of bronchiectasis was higher in patients with COPD than in the general population, and the risk was similar between men and women. The risk of bronchiectasis increased with age in COPD, and post-tuberculosis status was an important risk factor for bronchiectasis. The association between bronchiectasis and COPD is complex, and their cause and effect relationship, interaction, and systemic effects need to be further investigated.
